# Effect of multi-component school-based program on body mass index, cardiovascular and diabetes risks in a multi-ethnic study

**DOI:** 10.1186/s12887-019-1787-x

**Published:** 2019-11-04

**Authors:** Paula Costa-Urrutia, Rafael Álvarez-Fariña, Carolina Abud, Valentina Franco-Trecu, Julián Esparza-Romero, Cruz Mónica López-Morales, Martha Eunice Rodríguez-Arellano, Jaime Valle Leal, Valentina Colistro, Julio Granados

**Affiliations:** 1 Integrigen de Mexico SAPI de CV, 12 Patriotismo Avenue, No102. C.P. 06100. Hipódromo, Mexico City, Mexico; 2 Laboratorio de Medicina Genómica, Hospital Regional Lic. Adolfo López Mateos, ISSSTE, Universidad Avenue. P. C 01030, Álvaro Obregón, Florida, 0103Mexico City, 1321 Mexico, Mexico; 3 Departamento de Ecología y Evolución, Facultad de Ciencias, Universidad de la Republica, Montevideo, 4225 Iguá Street. P, C 11400 Montevideo, Uruguay; 4 Unidad de Investigación en Diabetes, Departamento de Nutrición Pública y Salud Coordinación de Nutrición, Centro de Investigación en Alimentación y Desarrollo, A. C, 46 Road Gustavo Enrique Astiazarán Rosas, La Victoria. PC, 83304 Hermosillo, Sonora Mexico; 5 Hospital General Regional 1, Instituto Mexicano del Seguro Social, Morelos. P.C.85110 Obregón, Ciudad Obregón Sonora, México, Sonora Mexico; 6 Coordinacion de Educación e Investigación en Salud, Hospital General Regional 1, Instituto Mexicano del Seguro Social, Morelos. P.C.85110 Obregón, Sonora, Mexico; 7 Departamento de Genética, Facultad de Medicina, Universidad de la República, 2125 General Flores Avenue. P.C, 11800 Montevideo, Uruguay; 8 División de Inmunogenética, Departamento de Trasplantes, Instituto Nacional de Ciencias Médicas y Nutrición Salvador Zubirán, 15 Vasco de Quiroga Avenue. P.C, 14080 Mexico City, Mexico

**Keywords:** Childhood obesity, Ethnic groups, BMI diabetes risk, Cardiovascular risk

## Abstract

**Background:**

Mexico occupies one of the first places worldwide in childhood obesity. Its Mestizo and Indigenous communities present different levels of westernization which have triggered different epidemiological diseases. We assessed the effects of a multi-component school-based intervention program on obesity, cardiovascular and diabetes risk factors.

**Methods:**

A physical activity, health education and parent involvement (PAHEPI) program was developed and applied in six urban (Mestizo ethnic group) and indigenous (Seri and Yaqui ethnic groups) primary schools for 12 weeks. A total of 320 children aged 4–12 years participated in intervention program; 203 under Treatment 1 (PAHEPI program) and 117, only from Mestizo groups, under Treatment 2 (PAHEPI+ school meals). For Body Mass Index (BMI), cardiovascular and diabetes factors, pairwise comparisons of values at baseline and after treatments were done using Wilcoxon signed rank test. Generalized linear models were applied to assess the intervention effect by age, sex and nutritional status in relation to ethnicity and treatment.

**Results:**

We observed improvements on BMI in children with overweight-obesity and in triglycerides in the three ethnic groups. The Mestizo ethnic group showed the largest improvements under Treatment 2. While Seris showed improvements only in cardiovascular risk factors, Yaquis also showed improvements in diabetes risk factors, though not in BMI.

**Conclusions:**

This study showed that the same intervention may have positive but different effects in different ethnic groups depending on their lifestyle and their emerging epidemiological disease. Including this type of intervention as part of the school curriculum would allow to adapt to ethnic group in order to contribute more efficiently to child welfare.

**Trial registration:**

This study was retrospectively registered under the identifier NCT03768245.

## Background

Childhood obesity has become one of the major health problems worldwide in the XXI century. Overweight and obesity increase cardiovascular disease and diabetes risks [1]. These diseases are rarely treated early in time, and consequently related morbility and mortality risks are increased [2].

The overweight-obesity prevalence in Mexico is one of the highest worldwide [3]; combined prevalence among schoolchildren between 5 and 11 years old is 34.4% [4]. Sonora State, in Mexico-United States border, shows the highest overweight-obesity prevalence in the country (36.9%) [5]. Signs of the typical food transformation of most industrialized countries are shown in Northern Mexico, among the Mestizo community and Indigenous ethnic groups, encompassing different levels of westernized lifestyle [6, 7]. The Seri ethnic group is the smallest population in the area; they have never exceeded one thousand inhabitants. Although they live in a natural environment, westernization permeates, changing traditional forms of nutrition and hydration [8]. This transition has triggered one of the highest diabetes prevalence in Mexico; 72% of the Seri community show diabetes in their family and 43% of adults developed prediabetes [8]. The Yaqui ethnic group is currently a population of 45,000 individuals divided in 8 villages in Northern Mexico, who live in rural and urban communities, and in Arizona, US [9, 10]. Adult Yaquis show the highest prevalence of overweight and obesity in northern areas (87.6%) [11]. In summary, such westernization has caused the typical health problems of the Western world, though differing in their emergence among ethnic groups [8, 11, 12].

Schools are regarded as a suitable setting for implementing obesity and related disease prevention programs, as they provide continuous and intensive contact with children, regardless of their ethnicity or socio economic status [13, 14]. However, the main issues concerning physical activity and nutrition arise in school children. In Mexico, physical education classes take place once a week and the effective time of physical activity is 10 min [15]. Unless nutrition programs are available at the school, food therein relies on home-made lunch and/or the snacks sold in kiosks inside the schools. These kiosks are in charge of vendors authorized by the school, but do not follow any nutritional requirement protocol. Hence, the school environment urgently needs to change the above mentioned situation, and thus developing childhood health programs in an educational context becomes necessary to address the obesity and related-disease problem.

We developed a school-based multi-component intervention program which included 60-min physical activity conducted five days a week, a health education workshop once a week, a meal serving program at the school five days a week and parent involvement activities to treat and prevent overweight, diabetes and cardiovascular risk in schoolchildren.

Thus, we evaluated the effect of the intervention on body mass index (BMI), total cholesterol (TC), low-density lipoprotein cholesterol (LDL); high-density lipoprotein cholesterol (HDL) triglycerides (TG), glucose (GL), glycosylated hemoglobin (Hb1Ac) in three ethnic groups with varying levels of westernization: Mestizos, Seris and Yaquis. In addition, we compared the effect between two treatments, with and without school meals, in the Mestizo community.

## Methods

We included children from one preschool and 5 primary schools (first to sixth grade), between 4 and 12 years old, from Sonora State, Northern Mexico. Four general urban schools of Mexican-Mestizos (from Hermosillo, capital city) and two indigenous schools, Seris (from Punta Chueca) and Yaquis (from Bahía de Lobos), participated in the program. Urban schools were blindly selected from a list of schools from Hermosillo provided by the Sonora Secretary of Education and Culture, while Indigenous schools were selected for being the most accessible ones. The intervention program was implemented from September to November 2016 (12 weeks, 60 business days). All children voluntarily accepted participating in the program, and their parents authorized their participation signing an informed consent. This project was approved by the Ethics Committee of Regional Hospital Lic. Adolfo López Mateos (Registry number 433.2016) from Institute for Social Security and Services for State Workers, México, and Sonora Education and Culture Secretary.

Intervention Components.

The intervention consisted of the following components: physical activity, health education, parent involvement and school meals.

### Physical activity component

Its objective was to develop an active scholar environment. It was implemented by a physical education teacher and consisted of a moderate-vigorous activity, five days a week. Physical activities were divided in two types (a) in the school backyard for 60 min, three times a week, and (b) in the classroom for 45 min (15 min three times a day), two days a week. Physical activity consisted of 20 circuits of strength, resistance, velocity and coordination, 10 circuits of stability and cardiovascular activity, and 10 circuits of pre-sport games. Complexity of circuits was adjusted for 3 levels: preschool, first to third grades, and fourth to sixth grades. Exercises and games were progressively intensified as tolerated by all children in the classroom.

### Health education component

Its objective was to empower children on healthy lifestyle, addressing four main topics: nutrition (i.e. healthy food, hydration), adequate quantities of food intake, physical activities and importance of self-monitoring (i.e. how to measure height and weight themselves). The program was implemented in the classroom through workshops planned and conducted by psychologists and nutritionists. Workshops were structured in three main parts: [1] triggering technique to raise interest in the topic, [2] experience-based technique by playing a game to comprehend, remember, and generate a change, and [3] triggering questions to generate analysis and reasoning on the topics addressed. Workshops lasted 50 min and were done once a week, for 12 weeks. Children were given a book with the four main topics addressed in this program.

### Parent involvement component

Parents participated in the intervention program through three workshops: 1) before the intervention, 2) after the first sampling, and 3) after the intervention program. They addressed the same four main topics as the workshops for children. Parents were given a report on results of the program and a guideline book with a straightforward link to that given to children, designed in order to enhance communication on adopting healthy habits.

### School meals

Its objective was to maintain lean mass and decrease fat mass as well as ensuring correct mineral, vitamins and fatty acids intake and adequate hydration for proper growth under National Specific Action Program: Food and Activity [16]. The source of total energy was distributed in a fat intake of 25–35%, a carbohydrate intake of 45–65% and a protein intake of 10–30%, while total calories were adjusted according to age. The Nutrition component consisted of three meals: breakfast, a snack for a mid-morning playtime, and lunch at school. Food was prepared daily following sanitary and quality standards of Mexican official regulation. Breakfast was served 40 min before class time, lunch was served 40 min before leaving school. Beverages consisted on milk in the morning and water in the rest of the servings. The teachers and our staff recorded attendance and controlled food intake of the children. Only the children who participated in at least 75% of the meals were taken into account for the analysis.

### Treatment implementation

Two treatments were applied. Treatment 1 (T1) consisted on physical activity, health education and parent involvement components, while treatment 2 (T2) consisted on physical activity, health education, parent involvement and school meals. Indigenous children received T1, whereas part of Mestizo children received T1 and T2. For T2 we worked with two grades, in three of the urban schools, in order to get a representative and homogenous sample of age (grades 1–2, 3–4, 5–6 each pair randomly selected from a different school). Anthropometric and blood samples were collected before and after treatments by the same team. All nutritionists, psychologists and physical education teachers received a two-week training course imparted by the developers of the project (PCU and RAF), who also supervised health education workshop and physical activity.

### Measurements

Weight was measured to the nearest 0.1 kg using a Seca scale. Height was measured to the nearest 0.1 cm using a standardized portable stadiometer Microtoise. Body Mass Index (BMI) was calculated as weight in kilograms divided by height in squared meters (kg/m^2^). BMI cut-off points of normal weight, overweight and obesity were used as defined by World Health Organization [17]. Blood samples were taken in the morning, after a 10-h fast to measure serum lipid profile (TC measured in mg/dl; LDL in mg/dl; HDL in mg/dl; and, TG in mg/dl) to assess cardiovascular risk, as well as glucose profile, GL in mg/dl and (Hb1Ac in %) to assess diabetes risk (Miura CO, LTD). The samples were kept in coolers no longer than two hours before further processing.

### Data analysis

We obtained descriptive results, as median and interquartile range of the primary outcomes (BMI, TC, LDL, HDL, TG, GL and HbA1c) analyzed by ethnicity. Within each ethnic group, and each treatment (T1 and T2), we performed pairwise comparisons of outcomes at baseline and after treatments using Wilcoxon signed rank test in XLSTAT software (Data Analysis and Statistical Solution for Microsoft Excel. Addinsoft, Paris, France 2017).

We calculated the outcome differences (Dif) as after-intervention minus baseline values for each measurement, obtaining DifBMI, DifTC, DifLDL, DifHDL, DifTG, DifGL and DifHb1Ac. To assess the effect of age, sex, ethnic group (Mestizos, Seris, Yaquis) and nutritional status (Normal weight, Overweight+Obesity) on each Dif we performed Generalized Linear Models (GLMs) with Gaussian distribution [18]. The effect of T2 was analysed independently performing another GLM. An initial model contained all single effects and all possible interactions of such explanatory variables. GLM simplification was done by stepwise deletion of the least significant terms. Subsequent models were generated by stepwise deletion and assessing each simplification with Akaike information criterion (AIC) using the ΔAIC> 2 criterion [19]. All models considered were subjected to customary residual analyses and showed a satisfactory fit (results not shown). All statistical analyses were carried out in R [20]. We performed a power analysis which allows to estimate the probability of detecting an effect of a given sample size with a given level of confidence, under sample size constraints. We estimated the power of each GLM at a significance level of 0.05; we included the effect size estimated by each GLM and the degrees of freedom according to each sample size (*n* = 320 for total dataset, *n* = 44 for mestizos under T1, *n* = 117 for mestizos under T2, n = 44 for Seris and *n* = 115 for Yaquis) minus the number of parameters estimates. For each GLM, power analysis was conducted using the pwr.f2.test command from the pwr package in R free software [21].

## Results

A total of 320 children, who followed the criteria to be included in the analyses, were sampled at baseline and after intervention (age x = 8.2 SD = 2.3, girls = 157, boys = 163). Conformation of the groups for analysis is shown in Table 1. At baseline, the highest medians of BMI, TC, LDL, TG and GL were found in Mestizos, while the lowest median of HDL and the highest median of HB1Ac were found in Seris (Table 1).

**Table 1:** Groups of girls and boys per ethnic group and treatments (T1 and T2) and the percentages of those classified with overweight-obesity (OWOB)

	Total number	Girls	Girls with OWOB	Boys	Boys with OWOB (%)
Mestizos under T1	44	12	7 (58.3%)	32	19 (59.3%)
Mestizos under T2	117	72	31 (43.1%)	45	21 (46.7%)
Seris	44	24	8 (33.3%)	20	8 (40%)
Yaquis	115	49	11 (22.4%)	66	21 (31.8%)

All descriptive values and comparisons at baseline and after intervention for T1 are shown in Table 2, and for T2 in Table 3. Overall, general results showed that Mestizos under T1 increased BMI, whereas those under T2 decreased it. Mestizo children improved values of TC, TG and GL significantly either if they were in T1 or in T2; however, the change in GL and TC was significantly higher under T2. Seris increased BMI, improved TC and TG values significantly, and increased GL and HB1Ac values significantly. Yaquis increased BMI significantly and improved TC, TG, GL and HB1Ac values significantly. The three ethnic groups showed an increase in LDL and a decrease in HDL.

**Table 2:** General characteristics and comparisons of the variables before and after treatment 1. Descriptive results related ethnic groups (Mestizos, Seris, Yaquis) at baseline and after treatments (AT) comparison. Outcomes were BMI (Body mass index), TC (Total Cholesterol), LDL (Low Density Lipoprotein), HDL (High Density Lipoprotein), GL (Glucose), HbA1c (Glycosylated hemoglobin). m (median), IQR (Inter Quartile Range, Q1-Q3). T1 consisted on Physical Activity Program, Health education program and Parent

Treatment 1									
	Mestizos			Seris			Yaquis		
Variables	Baseline m (IQR)	AT m (IQR)	p -value	Baseline m (IQR)	AT m (IQR)	*p*-value	Baseline m (IQR)	AT m (IQR)	p-value
BMI (km/m^2^)	16.8 (15.2, 19.8)	16.7 (15.2, 20.0)	0.94	17.6 (15.5, 20.5)	17.7 (15.4, 22.0)	0.84	16.1 (14.7, 18.7)	17.1 (15.5, 19.5)	9.0 × 10^− 3^
BMI z-score	0.3 (−0.4,1.8)	0.2(−0.5,1.1)	0.03	−0.4(− 0.8,0.6)	−0.5(− 0.8,0.3)	0.85	−0.4(− 0.8,0.1)	−0.4(− 0.7,0.4)	0.63
TC (mg/dl)	163.5 (141.5, 193.8)	162.5 (137.5, 179.7)	0.11	154.5 (134.5, 170.0)	134.4 (116.9, 150.2)	2.0 × 10^− 4^	147.0 (133.0, 162.0)	139.2 (118.8, 153.6)	1.0 × 10^− 4^
LDL (mg/dl)	76.1 (65.2, 96.6)	101.6 (83.1, 118.3)	1.0 × 10^− 4^	73.2 (64.0, 82.1)	94.1 (80.2, 106.5)	1.0 × 10^− 4^	75.0 (66.0, 83.5)	81.2 (66.8, 98.0)	9.0 × 10^− 3^
HDL (mg/dl)	61.0 (55.6, 68.3)	56.6 (51.7, 66.2)	0.23	53.7 (50.4, 59.2)	53.4 (47.7, 59.0)	0.16	55.0 (48.4, 59.8)	46.3 (40.3, 53.2)	1.0 × 10^− 4^
TGL (mg/dl)	91.0 (67.8, 131.8)	55.0 (40.8, 71.8)	1.0 × 10^− 4^	77.0 (61.0,103.5)	60.0 (43.0, 83.1)	6.0 × 10^− 3^	83.0 (59.0, 108.0)	60.0 (46.2, 83.6)	1.0 × 10^− 4^
GL (mg/dl)	89.9 (85.3, 93.3)	84.4 (79.8, 88.4)	4.0 × 10^−4^	80.0 (73.3, 84.0)	93.6 (88.2, 98.5)	1.0 × 10^− 4^	90.0 (86.0, 95.0)	84.9 (78.5, 89.8)	1.0 × 10^− 4^
Hb1AC (%)	5.4 (5.5,5.7)	5.4 (5.2, 5.7)	0.45	5.6 (5.5,5.7)	5.8 (5.6, 6.0)	1.0 × 10^−3^	5.5 (5.4, 5.6)	5.5 (5.2, 5.8)	0.38

involvement

**Table 3:** General characteristics and comparisons of the variables before and after treatment 2. Descriptive results from Mestizos ethnic group at baseline and after treatments (AT) comparison. Outcomes were BMI (Body mass index), TC (Total Cholesterol), LDL (Low Density Lipoprotein), HDL (High Density Lipoprotein), GL (Glucose), HbA1c (Glycosylated hemoglobin). m (median), IQR (Inter Quartile Range, Q1-Q3). T2 consisted on Physical Activity program, Health education program, Parent involvement and Nutrition program

Treatment 2			
Mestizos			
Variables	Baseline m (IQR))	AT m (IQR)	p-value
BMI (km/m^2^)	20.4 (16.8, 23.6)	21.1 (16.5, 23.7)	0.75
BMI z-score	0.07 (−0.5,0.6)	0.12 (− 0.6,0.5)	1.0 × 10^− 4^
TC (mg/dl)	171.5 (157.0, 193.5)	137.0 (121.2, 159.1)	1.0 × 10^− 4^
LDL (mg/dl)	90.0 (78.5, 104.5)	91.9 (78.2, 103.1)	0.25
HDL (mg/dl)	68.0 (60.0, 77.3)	54.6 (48.3, 63.9)	1.0 × 10^−4^
TGL (mg/dl)	91.0 (74.8, 133.0)	65.0 (50.0, 87.4)	1.0 × 10^− 4^
GL (mg/dl)	94.5 (87.0, 104.0)	81.8 (74.6, 86.9)	1.0 × 10^−4^
Hb1AC (%)	5.4 (5.3,5.5)	5.5 (5.2, 5.6)	0.14

Results of the effects of age, sex, ethnicity, and nutritional status on Dif values after T1, obtained by GLMs, are shown in Table 4. All minimum magnitude effect (β) showed more than 90% of statistical power, except for the minimum β in DifBMI (Age*Seris β = 0.04 *p* = 0.59) which showed 73%. In relation to ethnic group, Mestizos (β = − 0.35 *p* = 0.13) and Seris (β = − 0.57 *p* = 0.42) decreased their BMI values, but not significantly. Yaquis increased their BMI values (Yaquis β = 1.51 *p* = 1.0 × 10^− 4^ Table 4) and such amount of increase decreases with age (Age*Yaquis β = − 0.10 *p* = 0.02, Table 4). As regards nutritional status, a significant decrease was recorded for all children with Overweight-Obesity (β = − 0.22 p = 0.02 Table 4).

**Table 4:** Association results. Estimates (β),*p*-values and statistical power in percentage (Power %) for the minimum effect size (β) from the Generalized linear model of the effects of predictive variables, and the interaction between, them on the outcome differences between baseline and after intervention measurements: BMI (Body Mass Index), TC (Total Cholesterol), LDL (Low Density Lipoprotein), HDL (High Density Lipoprotein), GL (Glucose) Glycosylated hemoglobin (HbA1c). The explanatory variables were age, sex, ethnic group (Mestizos, Seris, Yaquis), and nutritional status (Normal weight, Overweight+Obesity)

Predictive Variables	β	P-value	Power (%)
Difference inBMI			
Intercept	−0.35	0.13	
Girls	− 0.15	0.09	
Age	0.06	0.04	
Seris	−0.57	0.42	
Yaquis	1.51	1 × 10^−4^	
Overweight-Obesity	−0.22	0.02	
Age*Seris	0.04	0.59	73
Age*Yaquis	−0.10	0.02	
Difference in TC			
Intercept	0.09	0.99	
Girls	−16.86	2.2 × 10^−4^	
Seris	21.98	0.38	
Yaquis	−30.39	0.02	
Age	−2.10	0.03	100
Girls*Seris	5.57	0.56	
Girls*Yaquis	16.60	0.02	
Age*Seris	−1.92	0.46	
Age*Yaquis	4.21	0.01	
Difference in LDL			
Intercept	5.03	0.02	
Seris	13.27	1.0 × 10^−4^	
Yaquis	2.22	0.34	100
Overweight-Obesity	5.27	0.02	
Girls	−4.09	0.05	
Difference in HDL			
Intercept	−6.14	6.6 × 10^−8^	
Girls	−4.03	0.01	
Boys*Seris	5.94	0.01	
Girls*Seris	8.21	2.6 × 10^−4^	
Boys*Yaquis	−2.60	0.11	100
Girls*Yaquis	2.60	0.13	
Difference inTGL			
Intercept	−34.50	2.0 × 10^−16^	
Seris	20.39	3.0 × 10^−3^	
Yaquis	18.95	1.7 × 10^−4^	
Overweight-Obesity	−9.99	0.03	100
Difference in GL			
Intercept	−12.30	2.0 × 10^− 16^	
EtniaSeris	26.90	2.0 × 10^− 16^	
EtniaYaquis	5.80	3.8 × 10^−05^	100
Difference in HbA1C			
Intercept	0.03	0.19	
EtniaSeris	0.10	4.0 × 10^−3^	99
EtniaYaquis	0.01	0.72	

Regarding cardiovascular risk factors, DifTC decreased mainly among girls (β = − 16.86, *p* = 2.2 × 10^− 4^), and in Yaquis (β = − 30.39, p = 0.02). In terms of DifLDL, the three ethnic groups showed an overall increase. As regards DifHDL, there was an overall decrease, except for Seri girls who showed a slight increase (β = 8.2 *p* = 2.6 × 10^− 4^).

A decrease in DifTG was recorded for the three ethnic groups, mainly in Mestizos (β = − 34.5 *p* = 2.0 × 10^− 16^), being greater in children with Overweight-Obesity of the three ethnic groups (β = − 9.99 *p* = 0.03).

Lastly, as regards diabetes factors, DifGL decreased in Mestizos and Yaquis, but increased in Seris (GL β = 26.9 p = 2.0 × 10^− 16^, HbA1c β = 0.10 *p* = 0.01 Table 3).

The effect of T2 in Mestizos improves almost all outcomes (Table 5). Improvements in DifBMI were marginally significant, while those on TC, LDL, HDL and GL were significant and no differences were found in TG. HbA1c slightly increased after T2. The lowest statistical power was 80%, detected in TG.

**Table 5:** Effect of Treatment 2 in Mestizos ethnic group. Estimates (β), p-values and statistical power in percentage (Power %) for the minimum effect size (β) from the Generalized linear model. Differences between outcomes analysed at baseline and after intervention: BMI (Body mass index), TC (Total Cholesterol), LDL (Low Density Lipoprotein), HDL (High Density Lipoprotein), GL (Glucose) Glycosylated hemoglobin (HbA1c). The explanatory variable was treatment 2

Outcomes	Variables	β	P-value	Power %
Difference in BMI	Intercept	0.09	0.44	
	T2	−0.25	0.08	99
Difference in TC	Intercept	−7.50	0.10	
	T2	−24.12	1.2 × 10^−05^	100
Difference in HDL	Intercept	−1.90	0.24	
	T2	−8.72	6.8 × 10^−06^	100
Difference in LDL	Intercept	21.81	1.1 × 10^−9^	
	T2	−22.24	1.6 × 10^−09^	100
Difference in TG	Intercept	−45.22	1.7 × 10^−10^	
	T2	7.99	0.30	80
Difference in GL	Intercept	−5.72	1.6 × 10^−3^	
	T2	−9.23	1.6 × 10^−05^	100
Difference in HbA1C	Intercept	−0.03	0.46	
	T2	0.08	0.08	93

## Discussion

We analyzed the effect of a multi-component intervention in three ethnic groups: Mestizos, Seris and Yaquis. Further, the effect of two different treatments was analysed in Mestizos. Overall, the results were heterogeneous among ethnic groups. First, we discuss similar results in the three groups studied, then we compare both treatments in Mestizos, and then we focus the discussion on Seris and Yaquis.

One of the most important results was that BMI decreased significantly in children with overweight and obesity, and that TG was improved in all children, especially in those with overweight and obesity in the three ethnic groups. Noteworthy, over 90% of statistical power was achieved in almost all minimum magnitude effects (except for one) in T1 and 80% in T2, thus the effect, or lack thereof, can be disregarded as being a statistical artefact.

Including all children avoids any stigmatization, and children with overweight and obesity are encouraged to make healthy changes when they are supported by changes in the schoolwide environment [22, 23]. The likely reason for the significant reduction in BMI among those children, as compared to non-overweight children, is that they presented a greater energy imbalance and, thus, greater adjustments were done in a healthy schoolwide environment by increasing vigorous physical activity and healthy food intake. Additionally, girls showed marginal greater improvements in BMI than boys. It is particularly important in girls, since they tend to deposit fat as part of their development in puberty, which tends to perpetuate to adulthood if they are in an obesogenic environment [24, 25].

The Mestizo groups showed improvements in TG and GL. Nevertheless, children under T2 showed improvements in almost all outcomes, except for TG which showed no differences and slightly increased HbA1C. Here, it is also important to note that the statistical power for TG comparisons was 80% while the remaining comparisons showed over 90% of statistical power.

HDL decreased significantly and LDL increased under T1; however, LDL decreased under T2 but we did not observe positive changes in HDL. It suggests that physical activity along with an education and school meals is effective for improving LDL in the short term, but improvements in HDL may take longer than 12 weeks. This is in line with previous intervention results indicating improved cardiovascular risk factors but no improvements in HDL [26]. In summary, Mestizo children under T2 significantly improved cardiovascular and diabetes risk factors, in line with evidence for multi-component interventions which encompass physical activity and nutrition programs showing better results in the short and long term [27, 28].

Mixed results were observed in Seri and Yaqui ethnic groups, which received just T1. Seris decreased their BMI (though not significantly) and factors of cardiovascular risk (TC and TG), though they increased their diabetes predictive values. Yaquis improved their values of cardiovascular (TC and TG) and diabetes risk factors (GL), but they increased their BMI. Differences in lifestyle and emerging health problems between both ethnic groups could explain, at least partly, these results.

Adult Yaquis show high prevalence of overweight and obesity (87.6%), diabetes (18.3%) [11] and hypertension (12%) [9]. These prevalence values are strongly linked with the high calorie intake from saturated fat, while high hypertension is linked to diabetes prevalence and central obesity. These chains of metabolic risks seem to start in the saturated fat intake, at least as one option [11].

In Yaqui school in Bahia de Lobos, though rural, food availability depends on external food vendors who are authorized by school authorities to sell foods to students during breaks, like in urban schools. These kiosks provide students with snack options rather than a full meal [29]. The snacks are usually cookies, peanut-based sweets or sweet rolls, while drinks are sugar sweetened whole milk or soda, and there is no control on the amount of intake. Even when children received the *Health education* program, they had no other options of food inside the school. This kiosk opened a month after the program started and intake of its food options increased as the program was conducted. Therefore, children increased their energy intake and had higher BMI values. The improvements observed in cardiovascular and diabetes risk factors were probably due to the higher physical activity intensity experienced during the program. Overall, acceptance from Yaquis to products high in saturated fats, typical of western lifestyle, also plays a major role in child population health.

Adult Seris show high prevalence of diabetes. A high proportion of individuals (72%) have a relative with diabetes. Prediabetes prevalence reaches 43% and that of overweight and obesity reaches 41% [8]. It is well-known that Seris have a high intake of soda partly because potable water lacks, and partly because it was culturally acquired. This high intake has driven to high diabetes incidence among adults of the community [8].

Seris show some differences from Yaquis and Mestizos in their food intake in school time. Seri children have lunch at home and come back to school, or a group of mothers cook for all children; thus, probably, homemade food intake is higher. We cannot rule out that the increase in physical activity may have led to an increase in soda intake, as a way of hydration for greater thirst. It could have contributed to an increase in GL and HbA1c levels. Overall, Seri community acceptance for soda intake instead of water as source of hydration plays a major role in child population health.

This study present limitations and advantages worth being noticed. One limitation could be that it was a short-term study and it may prevent us to observe changes especially in LDL, HDL and HB1Ac; the effect on these measures could take more than 12 weeks to be detectable under an intervention only in physical activity. Another limitation is that the study was not random and had no control group in indigenous ethnic group. It was so because these communities are hard to access. Acceptance is greater if the study implies a direct benefit to their children. Regarding urban schools, samples were taken and treatments were applied randomly, but because of practical reasons there was not a control group; however, it is a priority for further studies. To tackle this, we analyzed data by comparing treatments.

A major advantage of this study is that it does not present gaps between planning and conducting the program. It was highlighted the importance of researchers focus on the relation between program fidelity and programme results [30]. Physical education teachers, nutritionists, and psychologists were hired for this project and were supervised by the designers of the project (PCU and RAF).

## Conclusion

In conclusion, the study shows that a health intervention program can have positive effects on different ethnic groups, depending on lifestyles and epidemiologic factors affecting communities. The intervention was successful in reducing cardiovascular risk factors (TC and TG), and BMI in children with overweight and obesity. This is especially important in Mexico, a country in which the rise in childhood obesity and its related diseases is alarming. Thus, a higher understanding of massive treatments in an educational context is urgently needed and it is expected to impact on disease prevention and children health care. This is the first intervention study to assess obesity, diabetes and cardiovascular risk factors in three ethnic groups. It seems reasonable to suggest that this kind of intervention programs be part of the school curriculum. This kind of research would allow to reorder and adapt the intervention according to the environment, culture, and emerging health problems in each community, and thus to efficiently contribute to child welfare.

## Abbreviations

AIC: Akaike information criterion
BMI: Body Mass Index
Dif: Difference
DifM: Difference at any measurement
GL: Glucose
GLM: Generalized Linear Model
Hb1Ac: Glycosylated hemoglobin
HDL: High density lipoprotein cholesterol
LDL: Low density lipoprotein cholesterol
M1: Measurements 1
M2: Measurements 2
NP: Nutrition program
PAHEPI: Physical activity, health education and parent involvement
T1: Treatment 1
T2: Treatment 2
TC: Total cholesterol
TG: Triglycerides
